# Lightweight Structural Design of UAM Fuselage Using AI Predictive Modeling and Composite Big Data from Automated Manufacturing

**DOI:** 10.3390/ma19112222

**Published:** 2026-05-25

**Authors:** Woo Hyuk Son, Ji Hoon Kim, Sung-Youl Bae

**Affiliations:** 1 Aerospace Defense Research Group, Korea Institute of Ceramic Engineering & Technology, 101 Soho-ro, Jinju 52851, Republic of Korea; swh@kicet.re.kr; 2 School of Mechanical Engineering, Korea Pusan National University, Busandaehak-ro 63beon-gil, Geumjeong-gu, Busan 46241, Republic of Korea

**Keywords:** fiber reinforcement plastics (FRP), data-driven predictive model, urban air mobility (UAM), structural analysis, design process

## Abstract

**Highlights:**

- An AI-driven lightweight design framework integrating automated composite manufacturing and deep learning was established, reducing experimental dependency compared with conventional trial-and-error approaches.
- Under identical deformation conditions, composite fuselage structures achieved up to 50% weight reduction compared to aluminum while maintaining structural safety (IRF < 1).
- The developed deep learning regression model achieved R^2^ = 0.80 and a prediction error below 5%, demonstrating acceptable predictive capability for preliminary design screening within the investigated parameter space.
- Compared with traditional material-selection approaches, the proposed methodology enables data-driven stiffness optimization across multiple FRP systems.

**Abstract:**

Traffic congestion and air pollution caused by rapid urbanization have emerged as critical challenges in metropolitan areas worldwide. Urban air mobility (UAM), particularly electric propulsion-based systems, has gained attention as a promising solution. For the successful commercialization of UAM, a lightweight airframe design with ensured structural integrity is essential. This study proposes an optimized lightweight design process that integrates automated composite manufacturing with artificial intelligence (AI)-based material property prediction. Finite-element analysis (FEA) was performed on glass fiber-, basalt fiber-, and carbon fiber-reinforced polymers under identical deformation conditions to derive design material properties in terms of elastic modulus and weight reduction. A large-scale dataset of fiber-reinforced plastics was established through an automated manufacturing process, and a deep learning regression model was developed using Altair AI Studio to predict mechanical properties under untested material and process conditions. The predicted properties were applied to a UAM fuselage model, and FEA results demonstrated that composite structures achieved equivalent or superior stiffness with up to 50% weight reduction compared to aluminum. In addition, inverse reserve factor (IRF) analysis confirmed structural safety, with all configurations maintaining IRF values below 1. The proposed AI-driven framework provides a scalable and data-driven lightweight design methodology applicable to next-generation UAM and advanced air mobility structures.

## 1. Introduction

Rapid urbanization and population growth have intensified various urban issues such as traffic congestion and air pollution in large metropolitan areas. As an alternative solution, urban air mobility (UAM) has garnered significant attention, and research on eco-friendly, electrically powered UAM technologies has been actively conducted [1,2,3,4,5,6,7,8].

For the efficient and safe operation of UAM vehicles, both lightweight and structural durability must be achieved in the airframe design [9]. In conventional aircraft, the fuselage structure—excluding wings, propulsion, and tail components—constitutes 20–30% of the total structural weight, and the fuselage of UAM vehicles exhibits a similar ratio of 17–25% [10,11,12]. Therefore, reducing the fuselage weight is an effective strategy for minimizing the overall mass of a UAM vehicle [13,14].

Fiber-reinforced polymer (FRP) composites exhibit significantly higher specific stiffness and specific strength compared with conventional metallic materials. For example, CFRP can provide a stiffness-to-weight ratio approximately 3–5 times higher than that of aluminum alloys, depending on fiber orientation and volume fraction. This superior specific performance enables structural weight reduction without compromising load-bearing capacity [15,16,17,18,19].

Nevertheless, the variability of FRP mechanical properties can be statistically characterized by the coefficient of variation (CV), which typically ranges from 5% to 15% depending on fiber type, layup configuration, and manufacturing consistency. In conventional hand lay-up processes, reported CV values for tensile and flexural properties often exceed 10%, reflecting sensitivity to operator-dependent and process-induced variations [15,20,21,22,23]. These property variations can adversely affect the accuracy of design parameters, such as the strength and elastic modulus of composites, which are critical for reliable structural design. Consequently, recent studies have focused on introducing automated manufacturing processes to minimize such errors and improve production consistency [24,25,26,27].

Beyond manufacturing automation, recent advancements in artificial intelligence (AI) and deep learning have provided powerful tools to address the inherent variability of composite materials. Machine learning–based predictive models have been increasingly applied to estimate the mechanical properties of fiber-reinforced composites based on material composition and processing parameters [28,29,30]. These data-driven approaches enable rapid screening of material combinations and optimization of manufacturing conditions while significantly reducing experimental cost and development time.

In particular, deep neural network models have demonstrated strong capability in capturing nonlinear relationships between process variables and mechanical performance in composite systems. Such AI-assisted material design frameworks are now recognized as promising strategies for accelerating composite optimization in aerospace and advanced mobility applications [31,32].

In this study, important variables related to the material composition and forming conditions of FRPs are selected as input parameters, and identical specimens are fabricated using an automated composite manufacturing system to establish a large-scale database of mechanical properties. The acquired data are systematically managed and analyzed using Altair’s commercial AI Studio software (https://altair.com/altair-ai-studio), and a deep learning-based regression prediction model is developed. Subsequently, plate-level structural analyses of the metal and composite panels are performed to derive design material properties applicable to UAM fuselage structures, and the abovementioned prediction model is used to determine optimal conditions for achieving the desired mechanical performance. Finally, the predicted material properties are applied to a finite-element analysis (FEA) of a UAM fuselage to evaluate the structural integrity of the composite design. The ultimate goal of this study is to establish a systematic design and analysis process for the efficient lightweight structural design of UAM components by integrating automated manufacturing, data-driven modeling, and predictive simulation.

Despite significant advancements in composite materials and automated manufacturing technologies, an integrated design framework that systematically links structural stiffness derivation, large-scale manufacturing data, and AI-based predictive modeling for UAM fuselage structures remains insufficiently explored. Existing studies often focus either on material property enhancement or structural optimization independently, without establishing a closed-loop data-driven design methodology.

Therefore, the significance of this study lies in proposing a unified, stiffness-based AI-driven lightweight design framework that integrates automated composite manufacturing, database construction, predictive modeling, and fuselage-level structural validation. This approach moves beyond conventional material comparison and provides a scalable methodology for next-generation aerospace composite structures.

The structure of this paper is organized as follows: Section 2 introduces the proposed design framework and outlines its key components. Section 3 presents plate-level stiffness derivation and lightweight analysis. Section 4 describes database construction and deep learning-based predictive modeling. Section 5 validates the proposed framework through fuselage-level structural analysis. Finally, Section 6 summarizes the main findings and implications.

## 2. Design Process

This study aims to establish a component design process that is distinct from existing aerospace component design methods to achieve an efficient and systematic lightweight design for UAM and aircraft components. Therefore, this study proposes a new design process for securing the design properties and optimizing the lightweight design of FRP composites applicable to UAM fuselage structures.

Specifically, by quantitatively assessing the potential of FRPs as a substitute for metal materials, we established a composite design procedure that simultaneously achieves efficient lightweighting and structural integrity.

**Figure 1:**
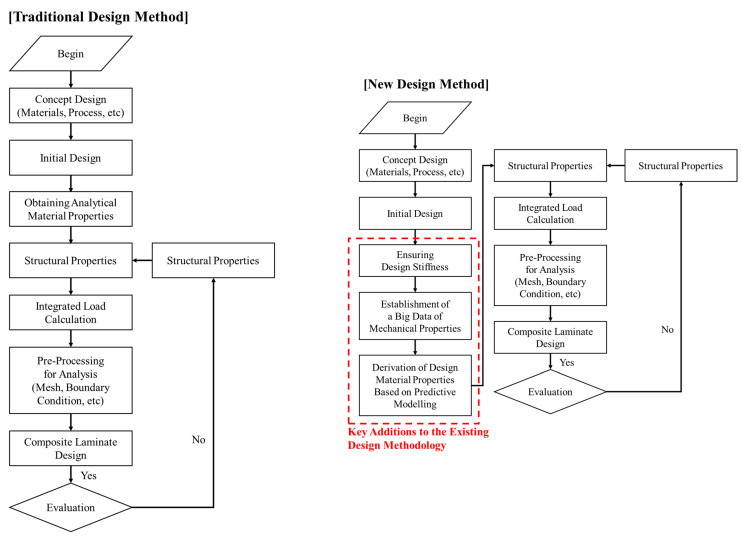
Comparison between existing and proposed composite component design processes.

**Figure 2:**
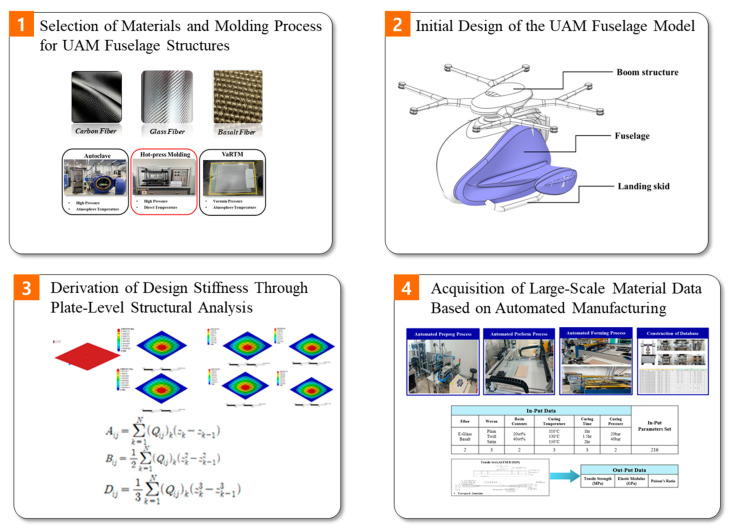

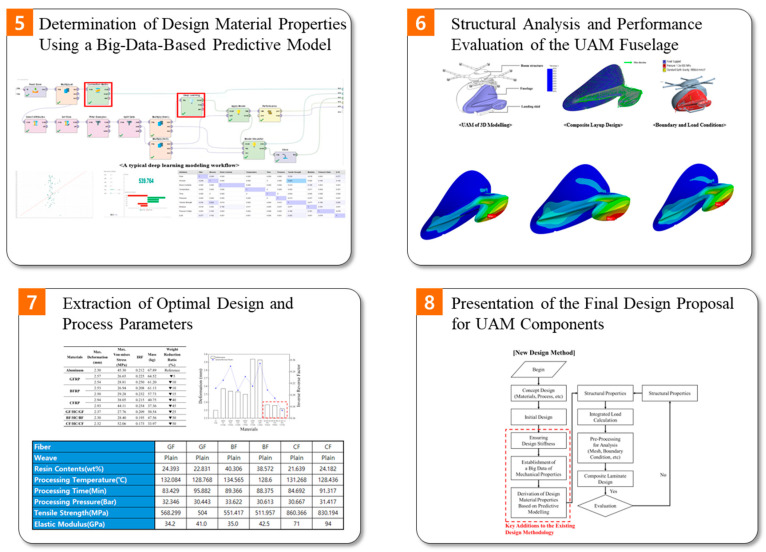
Schematic diagram of design process.

Unlike existing design methods, the proposed process derives the design stiffness through structural analysis at the plate level, which is then used to determine the required elastic modulus. Furthermore, through automated processes, we acquire large-scale material data and develop predictive models based on these data, thus enabling the effective estimation of process conditions and material properties that cannot be easily obtained experimentally. The composite component design process proposed in this study comprises the eight steps listed below, and Figure 1 shows a comparison between the existing composite component design process and the design process proposed in this study. Figure 2 presents a schematic diagram of the process.

1. Selection of materials and molding processes for the UAM fuselage structure.
2. Design of an initial UAM fuselage model.
3. Derivation of design stiffness through plate-unit structural analysis.
4. Acquisition of large-scale material data based on automated processes.
5. Derivation of design properties using a data-driven predictive model.
6. Analysis and performance review of the UAM fuselage structure.
7. Derivation of optimal design and process parameters.
8. Presentation of the final UAM component design.

As illustrated in Figure 2, the proposed design framework establishes a closed-loop integration between structural analysis, automated manufacturing, database construction, and AI-based predictive modeling. Unlike conventional composite design approaches that rely heavily on iterative experimental validation, the present framework derives target stiffness requirements through plate-level structural analysis and subsequently utilizes a trained deep learning model to identify optimal material compositions and processing parameters.

The predicted material properties are then directly applied to fuselage-level finite-element analysis, enabling rapid verification of structural integrity and lightweight performance. This integrated workflow not only minimizes trial-and-error experimentation but also provides a scalable methodology applicable to various aerospace composite structures.

Each subsequent section of this paper corresponds to specific stages of the framework presented in Figure 2. Section 3 addresses stiffness derivation through plate-level analysis, Section 4 describes database construction and predictive modeling, and Section 5 validates the proposed methodology through fuselage-level structural analysis.

## 3. Material Unit Lightweight and Stiffness Design

### 3.1. Material Unit Weight Reduction and Model Design

In this study, to quantitatively evaluate the lightweighting potential of a UAM fuselage structure, aluminum (Al 6061), which is widely used in conventional aircraft structures, was selected as the baseline material. The mechanical behavior and stiffness characteristics were analyzed when aluminum was replaced with various FRPs. The applied composite materials included glass fiber-reinforced plastic (GFRP), basalt fiber-reinforced plastic (BFRP), and carbon fiber-reinforced plastic (CFRP). The physical properties of each material, such as the density, elastic modulus, and tensile strength, were determined based on prior experimental data and the material database embedded in the commercial software ANSYS 2022 R2 version. The effective elastic modulus was estimated using the rule of mixtures, which approximates the longitudinal and transverse elastic properties based on fiber and matrix volume fractions [33]. The material properties of aluminum (Al 6061) were obtained from the ANSYS engineering data library. To ensure reliability, the adopted elastic modulus and Poisson’s ratio were verified against standard material property references. The ANSYS values (E ≈ 71 GPa, ν ≈ 0.33) are consistent with widely reported experimental data for Al 6061 in aerospace applications. For composite materials, orthotropic elastic constants were derived from experimentally validated plate-level stiffness results rather than relying solely on software library defaults. This approach improves consistency between material characterization and structural simulation.

The fuselage structure was simplified into a representative flat plate model to enable stiffness-based comparative evaluation under controlled boundary conditions. Such geometric idealization is commonly adopted in preliminary aerospace structural design, where local panel behavior is analyzed independently prior to full-scale assembly modeling. Since the primary objective of this study was to derive target stiffness under identical deformation constraints, the simplified plate model provides a controlled environment for isolating material-dependent stiffness effects. The thin-walled assumption is justified given the typical panel thickness-to-span ratio in aerospace fuselage structures.

To simplify the comparison of the mechanical behaviors of the materials, we employed plate-type models with a length-to-width ratio of 1:1 and maintained the dimensions at 100 mm × 100 mm. The thickness of each model was determined relative to a 3 mm thick aluminum plate and reduced accordingly to represent the desired degree of lightweighting for FRP application. Subsequently, plate-level structural analyses were performed to derive the effective elastic modulus of each material that would result in the same deformation as that of aluminum under identical loading conditions. The final thicknesses and corresponding material properties for each case are listed in Table 1. It should be emphasized that the 100 × 100 mm^2^ plate model with fully fixed edges and centrally applied load was employed solely as a stiffness calibration model. The objective was to derive equivalent elastic modulus values under identical deformation constraints for comparative material assessment. This simplified configuration does not represent the actual boundary conditions, load distributions, or curvature characteristics of real fuselage panels. Therefore, the plate-level analysis should be interpreted as a controlled stiffness equivalency evaluation rather than a realistic structural simulation of aircraft fuselage components.

**Figure 3:**
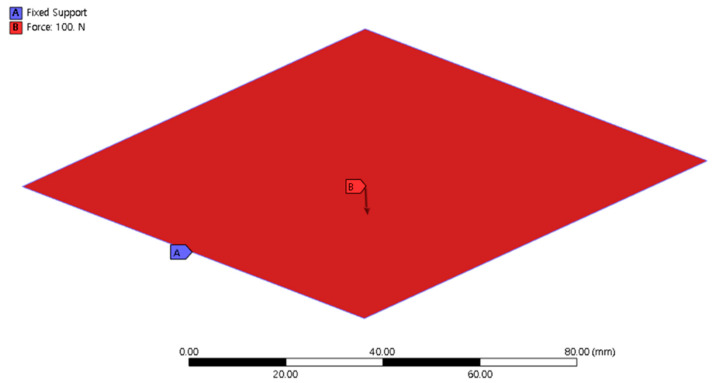
Boundary conditions applied to plate model.

The applied load magnitude (100 N) was selected as a representative static load to evaluate stiffness-based deformation behavior under controlled conditions. Since a linear elastic analysis was performed, deformation is directly proportional to the applied load. Therefore, the specific load magnitude does not affect the relative stiffness comparison among different materials. The primary objective was to compare material-dependent deformation under identical loading conditions rather than to simulate a specific operational load case. Accordingly, the selected load provides a consistent reference for stiffness-driven evaluation. As illustrated in Figure 3, fixed boundary conditions were applied along all four edges of the plate to prevent displacement. A static load of 100 N was uniformly applied along the vertical (z-axis) direction at the center of the plate’s upper surface. FEA was conducted using the commercial solver ANSYS, while the deformation, equivalent stress, and inverse reserve factor (IRF) of each model were compared to investigate the lightweighting efficiency and stiffness design performance of different FRP materials.

### 3.2. Results of Material-Level Structural Analysis

**Figure 4:**
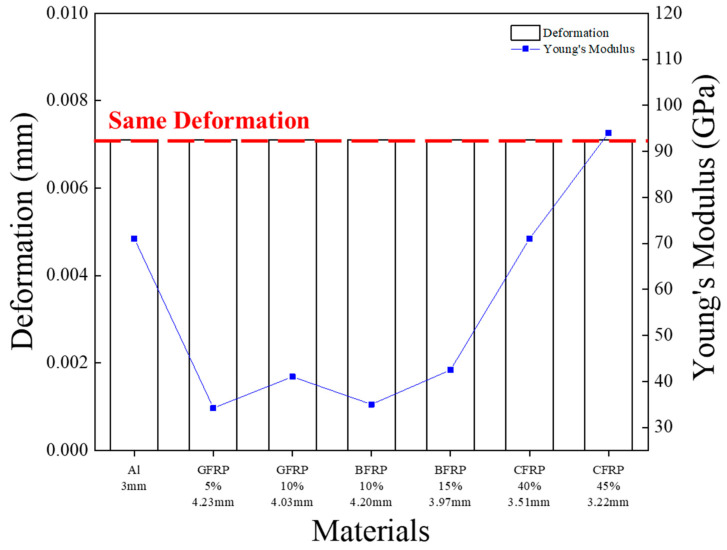
Structural stiffness analysis results of lightweight FRP.

In this study, FEA was performed using ANSYS to evaluate the structural behavior of plate-type models composed of aluminum, GFRP, BFRP, and CFRP. Simulations were conducted to determine the maximum deformation, equivalent stress, and elastic modulus corresponding to the identical deformation conditions for each material. As shown in Figure 4, the lightweighting ratio of the FRP plates was systematically varied from 5% to 45% relative to aluminum, and the corresponding mass and elastic modulus required to achieve the same maximum deformation were calculated. The results revealed a clear variation in structural stiffness with decreasing thickness when the composites were applied. Among the tested materials, CFRP exhibited the highest stiffness and lowest weight ratio, thus demonstrating the most effective lightweight performance. The derived elastic moduli were used as target values for the data-driven predictive model developed in the subsequent stage of this study.

## 4. Database Construction and Derivation of Design Variables

### 4.1. Acquisition of Material Data

In this study, an automated composite manufacturing system was designed to minimize human errors associated with manual operations and improve productivity in the iber–reinforced Plastics (FRP). Several challenges are encountered in conventional manual processes, including quality variations due to different operator skills, productivity degradation owing to repetitive tasks, and process errors occurring during forming operations. To quantitatively evaluate the impact of automation on process consistency, the statistical dispersion of mechanical properties was compared between manually fabricated and automatically manufactured specimens. For the manual fabrication group, the mean tensile strength was 415.2 MPa with a standard deviation of 10.45 MPa. For the automated manufacturing group, the mean tensile strength was 427.7 MPa with a standard deviation of 6.23 MPa. The standard deviation of tensile strength in the automated process was approximately 30% lower than that observed in manual fabrication. This reduction in variability indicates improved repeatability and reduced operator-dependent uncertainty. The improved consistency directly contributes to enhanced reliability of the constructed composite material database and subsequent predictive modeling.

**Figure 5:**
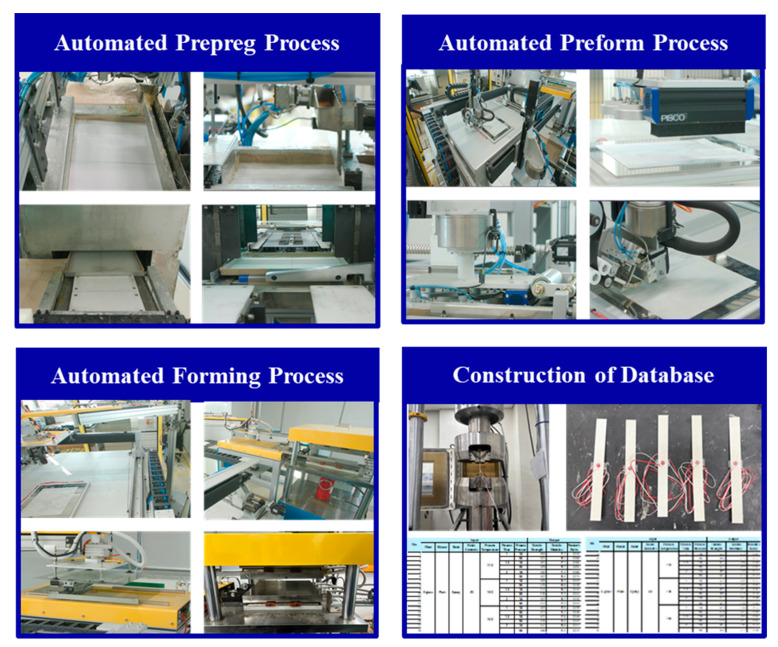
Process of establishing data for FRP.

Hence, an integrated automation system was developed that encompasses the entire manufacturing sequence from intermediate material preparation to final molding. Using this system, FRP with consistent and reliable quality was successfully fabricated. The automated manufacturing process was designed as a three-stage procedure comprising (1) intermediate material fabrication, (2) automated lay-up of intermediates, and (3) hot-press molding, as illustrated in Figure 5.

Mechanical-property evaluations, including tensile strength, elastic modulus, and Poisson’s ratio, were conducted in accordance with ASTM D3039 [34] to establish a reliable material-property database for the fabricated composites. To construct a comprehensive data repository, input variables such as the fiber type, fabric architecture, resin content, and molding parameters (pressure, time, and temperature) were selected. A total of 218 different manufacturing conditions were employed for the specimen fabrication. For each specimen, the tensile strength, elastic modulus, and Poisson’s ratio were defined as the output variables, which resulted in 3270 experimental data points. The constructed database served as a core training dataset for developing artificial intelligence (AI)-based predictive models and optimization algorithms for enhancing the design and processing efficiency of fiber-reinforced composites.

The constructed composite material database consists of structured input and output attributes related to material composition, manufacturing parameters, and measured mechanical properties. The input attributes include fiber type, weaving pattern, fiber volume fraction, resin type, curing temperature, curing pressure, and curing time. The output attributes correspond to experimentally measured mechanical properties, including tensile strength, flexural strength, elastic modulus, and interlaminar shear strength. Each data entry represents a unique combination of material composition and processing parameters, linked to corresponding mechanical test results. This structured database enables systematic correlation analysis and predictive modeling.

### 4.2. Deep Learning Model Architecture and Training Configuration

In this study, the mechanical properties of fiber–reinforced composites manufactured through an automated process were evaluated, and a corresponding dataset was constructed. Based on the established dataset, a deep learning–based prediction model was developed using AI Studio by Altair Co. to derive the target mechanical properties required for lightweight structural design. Altair AI Studio was selected as the predictive modeling platform due to its integrated industrial workflow compatibility and user-accessible machine learning environment. The primary objective of this study was not to develop a novel deep learning algorithm, but to establish a practical AI-driven design framework that can be readily adopted in industrial composite manufacturing environments. Compared with low-level open-source coding frameworks, AI Studio provides streamlined data preprocessing, correlation analysis, and regression modeling tools within a unified interface, facilitating rapid deployment and integration with engineering design workflows. The predictive model implemented in this study follows standard feed-forward neural network principles, which are reproducible using other machine learning platforms.

A total of 3270 experimental data points were used to train and evaluate the deep learning model. The dataset was randomly divided into training (70%), validation (15%), and test (15%) subsets. Accordingly, 2289 samples were used for training, 491 samples for validation, and 490 samples for testing. The data split was performed using random shuffling to ensure unbiased distribution across subsets. The test dataset was strictly excluded from the training process and was used solely for final performance evaluation to prevent data leakage. No data overlap occurred between the training, validation, and test sets.

**Figure 6:**
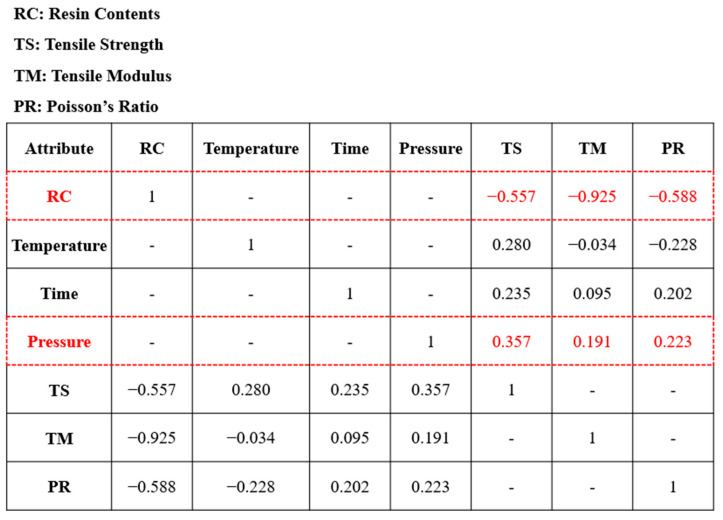
Correlation matrix of process parameters and mechanical properties.

To improve the predictive performance of the deep learning model, the correlation between input variables and output mechanical properties was analyzed, and variables with low influence were removed from the input set. The correlation matrix is presented in Figure 6. In the matrix, coefficients close to −1 indicate a strong negative correlation, values near +1 represent a strong positive correlation, and values close to zero imply little to no correlation.

Based on the derived correlation matrix, the influence between process parameters and mechanical properties was assessed. The results revealed that resin content and molding pressure exhibited relatively strong effects on tensile strength and elastic modulus. Resin content showed negative correlations of −0.557 for tensile strength and −0.925 for elastic modulus, indicating that both properties increase as the resin content decreases. Conversely, molding pressure presented positive correlations of 0.357 for tensile strength and 0.191 for elastic modulus, suggesting that higher molding pressure tends to improve mechanical performance. Meanwhile, molding time and molding temperature exhibited relatively low correlation coefficients for both properties, implying limited influence under the processing conditions considered in this study. These findings were then used to remove low-importance variables during model training, thereby improving prediction accuracy.

Prior to model development, correlation analysis was conducted to evaluate the independence of input variables. Pearson correlation coefficients were calculated among all candidate input parameters. Variables exhibiting strong linear correlation (|r| > 0.95) were carefully examined to avoid multicollinearity effects. Redundant or highly correlated variables were either removed or retained only when physically justified. This preprocessing step ensured that the input features used in the deep learning model were sufficiently independent to prevent instability or bias in regression performance.

To ensure the reproducibility and reliability of the proposed predictive model, the architecture and training configuration of the deep learning regression model are described in detail in this section.

The predictive model was implemented using Altair AI Studio and constructed as a fully connected feed-forward neural network. Based on preliminary sensitivity analyses and learning stability considerations, the network architecture was composed of two to three hidden layers, with node configurations of 128, 64, and 32 neurons, respectively. The Rectified Linear Unit (ReLU) activation function was employed for all hidden layers to efficiently capture nonlinear relationships between processing parameters and mechanical properties while mitigating gradient vanishing issues.

The output layer consisted of linear activation functions to predict continuous mechanical properties, including tensile strength and elastic modulus. Model training was conducted using the Adam optimizer, which was selected due to its robust convergence behavior for nonlinear regression problems. The mean absolute error (MAE) was adopted as the loss function, as it provides stable performance against outliers and directly reflects prediction accuracy in engineering applications.

The learning rate was set to 0.001, and the batch size was fixed at 32 based on convergence stability observed during preliminary training trials. The total number of training epochs was set to 200, which ensured sufficient convergence without overfitting.

**Figure 7:**
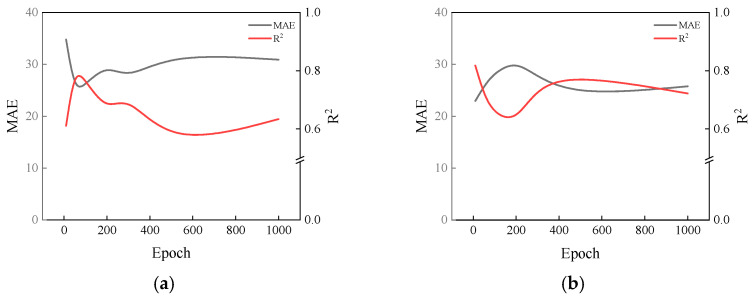

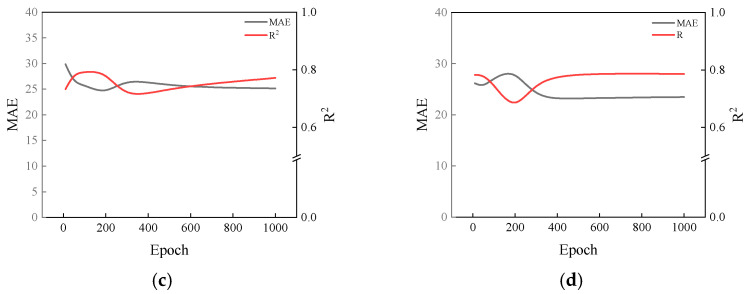
Model performance according to the number of hidden layers. (**a**) Number of Hidden layer: 1; (**b**) Number of Hidden layer: 2; (**c**) Number of Hidden layer: 3; (**d**) Number of Hidden layer: 4.

To evaluate the generalization capability of the model, the dataset was randomly divided into training (70%), validation (15%), and test (15%) subsets. The validation dataset was used to monitor learning convergence and prevent overfitting, while the test dataset was reserved exclusively for final performance evaluation. Overfitting behavior was assessed by comparing training and validation MAE trends, as illustrated in Figure 7.

This architecture and training configuration were selected to balance prediction accuracy, computational efficiency, and model interpretability, ensuring that the proposed model can be reliably reproduced and extended in future composite material design studies.

Although the predictive performance of the proposed model was quantitatively evaluated using RMSE, MAE, and R^2^ metrics, additional considerations were made to assess its generalization capability beyond simple statistical fitting.

First, the dataset was explicitly divided into training, validation, and test subsets, and the reported performance metrics were obtained from the test dataset, which was not used during model training. This approach ensured that the evaluation reflects the model’s predictive reliability on unseen data rather than memorization of existing experimental samples.

Second, the primary objective of the proposed model is not to reproduce measured data under identical experimental conditions, but to predict mechanical properties for untested combinations of material constituents and processing parameters. In this study, the trained model was successfully applied to identify material compositions and molding conditions that satisfy target elastic moduli derived from plate-level structural analysis, even when such combinations were not included in the original experimental dataset (Table 2).

Furthermore, overfitting behavior was carefully monitored through the comparison of training and validation learning curves. As shown in Figure 7, the model exhibited stable convergence with minimal divergence between training and validation errors when using two to three hidden layers, indicating robust generalization behavior.

Considering the inherent variability and nonlinear characteristics of fiber-reinforced composites, the achieved prediction accuracy (R^2^ = 0.80 and relative error < 5%) demonstrates that the proposed model provides sufficient reliability for design-oriented material screening and lightweight structural optimization, rather than purely statistical curve fitting.

It should be noted that experimental composite datasets inherently contain measurement uncertainty and process-induced variability. Such uncertainty may influence the accuracy of predictive modeling. In this study, data variability was partially reflected through statistical dispersion within the dataset, and the model was trained on randomly shuffled data to improve generalization robustness.

Although the present deep learning framework captures nonlinear relationships between input parameters and mechanical properties, explicit uncertainty quantification was not incorporated. Future research may integrate probabilistic modeling or Monte Carlo simulation to evaluate the sensitivity of predictions to input data uncertainty.

To further evaluate statistical robustness, residual analysis was conducted on the independent test dataset. The residuals exhibited a near-zero mean value, indicating the absence of systematic prediction bias. The standard deviation of the residuals remained small relative to the full mechanical property range, confirming stable prediction behavior within the investigated parameter space. The reported ±5% error corresponds to the mean absolute percentage error (MAPE) calculated over the independent test dataset.

### 4.3. Data-Driven Predictive Analysis

In this study, the mechanical properties of FRP through an automated manufacturing process were experimentally evaluated, and a corresponding large-scale data repository was systematically constructed. The automated process enabled the acquisition of consistent and high-reliability material data by minimizing operator-dependent variability and ensuring stable control of processing parameters. Based on this dataset, a deep learning-based predictive model was developed using Altair’s commercial software, AI Studio, to support data-driven determination of target material properties required for lightweight structural design.

The proposed predictive model was designed to estimate the mechanical properties of unmeasured material combinations and molding conditions that were not experimentally tested, thereby overcoming the limitations associated with conventional trial-and-error experimental approaches. Using this model, material compositions and processing parameters that satisfy the target elastic modulus derived from plate-level structural analysis were successfully identified. The predicted material properties and corresponding process conditions are summarized in Table 2.

The outcomes of this predictive analysis provide fundamental reference data for the lightweight structural design and numerical simulation of UAM fuselage components. Furthermore, the proposed data-driven approach is expected to enhance design efficiency by reducing experimental cost and development time, while offering scalability for application to various composite-based aerospace and advanced mobility structures in future studies.

## 5. Material-Level Lightweight and Stiffness Design

### 5.1. UAM Fuselage Structural Design

In the finite element analysis, aluminum (Al 6061) was modeled as an isotropic linear elastic material. In contrast, the fiber-reinforced polymer composites (GFRP, BFRP, and CFRP) were modeled as orthotropic materials, reflecting their direction-dependent mechanical behavior (Table 3). The orthotropic elastic constants (E_1_, E_2_, G_12_, ν_12_) derived from the plate-level stiffness design were assigned to the composite laminates. This approach ensures that the anisotropic characteristics of the composite materials are appropriately represented in the structural simulations.

### 5.2. Boundary Conditions for UAM Fuselage Structure

For the optimal structural design of the UAM fuselage, an analysis was conducted under flight conditions corresponding to a maximum flight altitude of 300 m and a maximum cruising speed of 22 m/s. Additionally, a rear gust velocity of 9 m/s, as specified in the Aviation Technical Standards Part 27, was considered to estimate the maximum dynamic pressure exerted on the fuselage during flight.

The dynamic pressure (q) applied to the fuselage surface was calculated using Equation (1), where the cruising speed ($V_{1}$) was 22 m/s, and the gust velocity ($V_{2}$) was −9 m/s. Based on these parameters, the pressure exerted on the fuselage surface was calculated to be 0.6 kPa. To account for potential disturbances and uncertainties encountered during an actual flight, a safety factor of 2, which is commonly adopted in the aerospace industry, was applied. Consequently, the final design load condition was set to 1.2 kPa. Here, P denotes the pressure, $V_{2}$ represents the velocity of the incoming tailwind, and $V_{1}$ indicates the cruising speed of the UAM.

**Figure 8:**
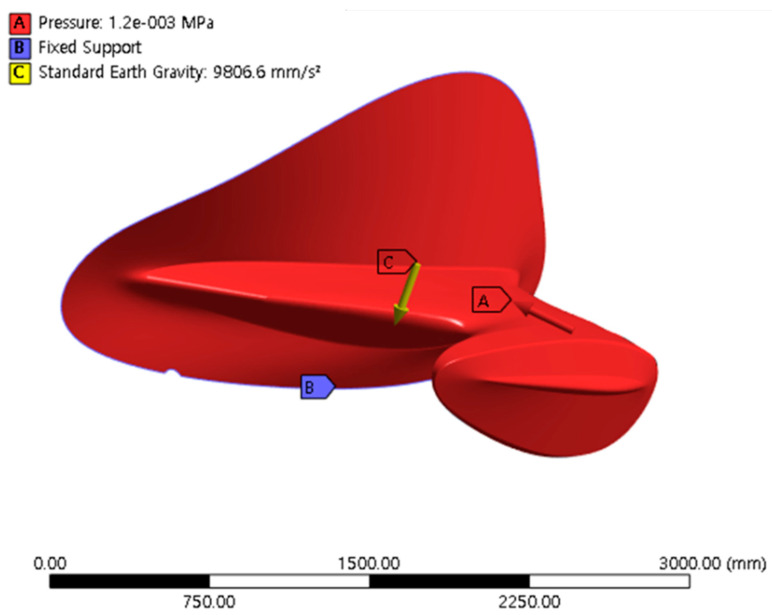
Boundary conditions applied to UAM fuselage model.

Regarding the boundary conditions, fixed supports were applied at the central fuselage section, where the UAM boom structure and propulsion system were mounted, and at the lower fuselage region connected to the landing skids. The complete configurations of the boundary and loading conditions applied to the fuselage model are illustrated in Figure 8.

(1) $$P={{0.5x(V}_{2}-V_{1})}^{2}$$

The boundary conditions and pressure load were selected to represent a simplified fuselage loading scenario for comparative evaluation purposes. The primary objective of this study was to assess relative structural performance among different material configurations under identical loading constraints. A full sensitivity analysis of boundary conditions was not conducted in the present study. However, since all material configurations were evaluated under the same boundary and loading conditions, the comparative weight reduction and structural feasibility trends remain valid. Future work may incorporate parametric sensitivity analysis to investigate the influence of varying boundary constraints and load magnitudes.

### 5.3. Results of Structural Analysis

The proposed design framework incorporates explicit decision criteria at each stage. First, the target stiffness was determined such that the maximum deformation under identical loading conditions did not exceed that of the aluminum reference structure. Second, predicted material configurations satisfying the target modulus within a predefined tolerance (±5%) were shortlisted. Finally, structural validation was performed based on two criteria: (i) inverse reserve factor (IRF) less than 1.0 and (ii) deformation limits comparable to the baseline model. In the case of composite materials, the allowable stress was determined based on the Puck failure index, where IRF corresponds to the maximum failure index under the applied load. IRF < 1.0 indicates no failure initiation.

Structural analyses were performed by applying Aluminum, GFRP, BFRP, and CFRP materials to the UAM fuselage structure using Ansys simulations. The previously derived design material properties were applied as input parameters for the structural analysis. Additionally, to achieve further weight reduction, a finite element model of a sandwich structure incorporating a honeycomb core was developed. The maximum deformation observed in all configurations remained sufficiently small compared to the structural dimensions, validating the small-strain assumption and justifying the use of linear analysis. The design of the honeycomb-core sandwich structure was determined considering core thickness and the corresponding weight reduction. Although increasing the core thickness provides advantages such as higher weight reduction and reduced deformation, the design was constrained to avoid interference with adjacent aircraft components.

**Figure 9:**
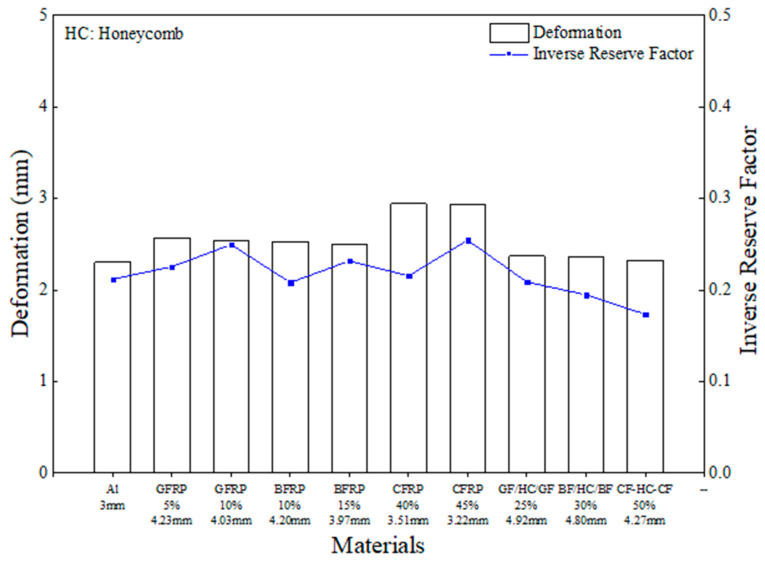
Structural analysis results of UAM fuselage with lightweight materials applied.

Including the sandwich structures, a total of ten material configurations were analyzed to evaluate the weight, thickness, maximum deformation, weight reduction ratio, and the failure index represented by the IRF (Inverse Reserve Factor). The results are summarized in Figure 9. Different failure criteria were adopted for metals and composite materials due to their fundamentally different mechanical behaviors. For aluminum, the von Mises yield criterion was applied, as it is appropriate for isotropic ductile metals.

For composite materials, the Puck failure criterion was employed to account for anisotropic behavior and distinct fiber and inter-fiber failure modes. Applying identical failure criteria to both material systems would not accurately represent their physical failure mechanisms. Therefore, material-specific failure models were selected to ensure a physically meaningful structural safety evaluation.

The analysis showed that the deformation differed by approximately 10% when only a single material was applied, which is attributed to analytical variation caused by differences in structural geometry and boundary conditions. In contrast, when applying the 10% lightweight elastic modulus of the composite materials to the honeycomb sandwich structure, the resulting deformation was similar to that of Aluminum. For composites, the Puck failure theory was applied, whereas for metallic materials, a yield-stress-based safety factor was used to evaluate structural integrity.

In this study, different failure criteria were employed for composite and metallic materials to appropriately reflect their distinct mechanical failure mechanisms, while a unified inverse reserve factor (IRF) was used as a common structural safety index.

For composite materials, the Puck failure criterion was adopted, as it is widely recognized for its capability to distinguish between fiber-dominated failure and inter-fiber fracture modes, which are critical in anisotropic laminated composites. The key parameters required for the Puck criterion, including longitudinal and transverse tensile and compressive strengths as well as in-plane shear strength, were determined based on experimentally measured material properties and reference values reported in the literature [35,36,37]. In addition, fracture plane inclination parameters and interaction coefficients were adopted from standard formulations of the Puck theory and implemented using the default settings provided in the commercial finite element software ANSYS, which have been validated in numerous composite structural studies.

For metallic materials, a yield-based failure criterion was employed, as plastic yielding governs the onset of failure in isotropic ductile metals such as aluminum alloys. In this case, the reserve factor was evaluated based on the ratio between the applied equivalent stress and the material yield strength.

Although different failure theories were used, the physical interpretation of IRF < 1 remains consistent across material systems, representing that the applied stress state does not exceed the allowable strength or failure envelope of the corresponding material. Therefore, IRF serves as a unified, non-dimensional safety index that enables direct comparison of structural integrity between composite and metallic configurations, even when different failure mechanisms are involved.

The use of IRF as a common evaluation index for hybrid material systems has been widely adopted in aerospace composite structural analyses, as it provides a conservative and transparent measure of failure margin while accommodating material-specific failure theories.

The IRF results indicated that single composite structures exhibited values 5–10% higher than Aluminum. However, when the honeycomb core was introduced, the IRF values became even lower. In all cases, the IRF remained below 1, confirming that no failure occurred and that structural integrity was fully ensured. Notably, the application of the honeycomb structure enabled up to approximately 50% weight reduction. The honeycomb sandwich configuration demonstrated up to approximately 50% mass reduction relative to the baseline aluminum model within the same numerical configuration. This result represents a model-level comparative outcome under simplified loading and boundary conditions. These findings demonstrate that the composite-based UAM fuselage design process proposed in this study demonstrates the lightweight potential of composite sandwich configurations within a stiffness-controlled numerical design framework. while maintaining structural stability.

It should be noted that the structural validation performed in this study is limited to static load analysis under simplified boundary conditions. The IRF-based evaluation confirms the absence of instantaneous failure initiation according to the selected failure criteria. However, the present analysis does not account for fatigue behavior, buckling stability, impact damage tolerance, progressive failure mechanisms, interlaminar delamination, or aeroelastic coupling effects. These factors are critical for certification-level aircraft structural validation and require further investigation. Therefore, the current results should be interpreted as a conceptual structural feasibility assessment within a stiffness-driven comparative design framework rather than a comprehensive airworthiness certification analysis.

## 6. Conclusions

In this study, plate-level structural analyses were performed to derive the design material properties of composites applied to a UAM fuselage structure. A data-driven AI predictive system was established to determine the optimal material and processing conditions for satisfying the required design properties. Furthermore, the predicted mechanical properties were applied to a UAM fuselage structure model to evaluate its structural integrity through FEA, thereby establishing a comprehensive lightweight design process for UAM structures.

- Based on plate-level structural analysis, we confirmed that GFRP and BFRP composites exhibited superior lightweight and stiffness characteristics compared with conventional aluminum. Under identical deformation conditions, the composites achieved up to 10% weight reduction relative to aluminum.
- An AI-driven predictive model was developed based on a big-data repository obtained from the automated manufacturing of CFRCs. This model effectively predicted material and process conditions that satisfy the target mechanical properties, even for combinations not considered in prior experiments.
- Applying the predicted material properties to the UAM fuselage structural analysis demonstrated that the honeycomb sandwich configuration achieved up to 50% weight reduction while maintaining an IRF of less than 1.0, thereby ensuring structural integrity and damage resistance.
- It is important to emphasize that the comparison among GFRP, BFRP, and CFRP was not intended to identify an absolutely superior material. Rather, this comparative evaluation was conducted to validate the robustness and applicability of the proposed stiffness-based AI-driven design methodology across composite systems with diverse mechanical characteristics and economic trade-offs. Overall, the composite configurations demonstrated competitive structural performance compared with conventional metallic structures, confirming their feasibility for next-generation UAM applications.
- The composite-based lightweight design process proposed in this study offers high scalability and practicality for future mobility-structure designs, including UAM platforms. This paper presents an effective and reliable approach for achieving lightweight structural optimization while maintaining safety and performance in next-generation aerospace systems.

Beyond the numerical results, the key contribution of this study lies in establishing an integrated, data-driven lightweight design framework that connects automated composite manufacturing, database construction, deep learning-based predictive modeling, and structural validation within a unified workflow. Unlike conventional trial-and-error material substitution approaches, the proposed methodology enables stiffness-driven material selection under identical deformation constraints, thereby improving design efficiency and scalability. The framework demonstrated material independence and can be extended to other composite systems and structural components in future aerospace and advanced mobility applications. Therefore, this study contributes not only to lightweight fuselage design but also to the broader development of AI-assisted composite structural optimization strategies.

## Figures and Tables

**Table 1:** Applied material and modeling information.

Materials	Thickness (mm)	Mass (g)	Weight-Reduction Ratio (%)
Aluminum	3.000	83.10	Reference
GFRP	4.251	78.95	5
	4.027	74.79	10
BFRP	4.202	78.95	10
	3.968	74.79	15
CFRP	3.511	49.86	40
	3.219	45.70	45

**Table 2:** Results of predictive value using an AI predictive model.

Fiber	GF	GF	BF	BF	CF	CF
Weave	Plain	Plain	Plain	Plain	Plain	Plain
Resin Content (wt.%)	24.4	22.8	40.3	38.6	24.1	21.6
Processing Pressure (bar)	30.4	32.3	30.6	33.6	30.7	31.4
Tensile Strength (MPa)	568.3	504.0	551.4	512.0	860.4	830.2
Elastic Modulus (GPa)	34.2	41.0	35.0	42.5	71.0	94.0

**Table 3:** Results of stacking design.

Materials	Thickness (mm)	Total Ply	Weight-Reduction Ratio (%)
Aluminum	3.000	1	Reference
GFRP	4.251	20	5
	4.027	20	10
BFRP	4.202	20	10
	3.968	20	15
CFRP	3.511	20	40
	3.219	20	45

## Data Availability

The original contributions presented in this study are included in the article. Further inquiries can be directed to the corresponding authors.
